# The role of prednisolone in acute urticaria management

**DOI:** 10.1186/2045-7022-5-S1-O8

**Published:** 2015-03-11

**Authors:** Kiran Godse

**Affiliations:** 1 Patil hospital, Navi Mumbai, India

## Aim

To evaluate the efficacy of a 5 day short course of oral prednisolone when added with levocetirizine for management of acute urticaria.

## Materials and methods

Prospective, randomized clinical trial was carried out in a teaching hospital.

All patients were asked to evaluate the severity of pruritus on urticarial activity score (UAS).

Patients were then given oral prednisolone 30 mg for 5 days and tablet levocetirizine 5 mg twice daily for 6 weeks and only levocetirizine tablet 5 mg twice daily for 6 weeks. Patients’ conditions were reassessed clinically with UAS calculated again 2 days later and again 5 days later.

## Results

49 patients were enrolled; 24 patients received prednisolone with Levocetirizine and 25 received only Levocetirizine. The two groups had similar UAS at enrollment (prednisolone, 4.6 ; levocetirizine ,4.4 ) , but at 2- and 5- day follow-up the prednisolone group had significantly lower UAS (1.4 and 0.2 versus 3.8 and 2.4 respectively) and greater clinical improvement in rash. Response did not correlate with age, sex, or identification of an allergen. No adverse effects were noted in levocetirizine group. In Prednisolone group two patients complained of gastritis.

## Conclusion

At the end of 6 weeks, 3 patients from the steroid group and 8 patients from the levocetirizine group continued to get urticarial wheals. The addition of a prednisolone short course improves the symptomatic and clinical response of acute urticaria to antihistamines. Patients with steroids improved more quickly and completely without major adverse effects.

